# The Giant Adhesin SiiE of *Salmonella enterica*

**DOI:** 10.3390/molecules20011134

**Published:** 2015-01-12

**Authors:** Britta Barlag, Michael Hensel

**Affiliations:** Abteilung Mikrobiologie, Fachbereich Biologie/Chemie, Universität Osnabrück, Barbarastr. 11, Osnabrück 49076, Germany; E-Mail: Britta.Barlag@biologie.uni-osnabrueck.de

**Keywords:** SiiE, bacterial IG domain, non-fimbrial adhesin, type I secretion system, lectin-like adhesin

## Abstract

*Salmonella enterica* is a Gram-negative, food-borne pathogen, which colonizes the intestinal tract and invades enterocytes. Invasion of polarized cells depends on the SPI1-encoded type III secretion system (T3SS) and the SPI4-encoded type I secretion system (T1SS). The substrate of this T1SS is the non-fimbrial giant adhesin SiiE. With a size of 595 kDa, SiiE is the largest protein of the *Salmonella* proteome and consists of 53 repetitive bacterial immunoglobulin (BIg) domains, each containing several conserved residues. As known for other T1SS substrates, such as *E. coli* HlyA, Ca^2+^ ions bound by conserved D residues within the BIg domains stabilize the protein and facilitate secretion. The adhesin SiiE mediates the first contact to the host cell and thereby positions the SPI1-T3SS to initiate the translocation of a cocktail of effector proteins. This leads to actin remodeling, membrane ruffle formation and bacterial internalization. SiiE binds to host cell apical membranes in a lectin-like manner. GlcNAc and α2–3 linked sialic acid-containing structures are ligands of SiiE. Since SiiE shows repetitive domain architecture, we propose a zipper-like binding mediated by each individual BIg domain. In this review, we discuss the characteristics of the SPI4-T1SS and the giant adhesin SiiE.

## 1. Introduction

### Salmonella Enterica Pathogenicity

*Salmonella enterica* is a food-borne pathogen that is able to infect a broad range of hosts and colonizes various niches in infected hosts [1]. As many other pathogens, *Salmonella* possesses a large number of virulence factors, which are mostly encoded by so-called Pathogenicity Islands. Depending on the serotype of *S. enterica*, up to 19 *Salmonella* Pathogenicity Islands (SPI) have been described [2,3], with SPI1 and SPI2 being studied in more detail. The SPI1-encoded type III secretion system (T3SS) functions are required for invasion of non-phagocytic cells by *Salmonella*. The SPI1-encoded T3SS translocates into host cells a cocktail of effector proteins leading to actin remodeling, membrane ruffling and finally uptake of *Salmonella*. SPI2 also encodes for a T3SS, but this system is responsible for the intracellular survival and proliferation of *Salmonella* inside host cells (reviewed in [1]). After invasion or phagocytic uptake, *Salmonella* resides in a modified phagosome, the *Salmonella-*containing vacuole (SCV), in which *Salmonella* is able to survive and replicate [4]. The SPI2-T3SS translocated effector proteins are responsible for the biogenesis of this organelle. Over time, the markers of the SCV membrane turn from early endosomal proteins to late endosomal proteins. Besides maintaining the SCV, SPI2-T3SS effector proteins are responsible for induction of tubular membrane vesicles, termed *Salmonella-*induced filaments (SIF).

To establish intimate contact to, and subsequently invade polarized epithelial cells, *Salmonella* additionally requires the function of the SPI4-encoded type I secretion system (T1SS) and its substrate SiiE. In contrast, invasion of non-polarized cells is completely SiiE-independent [5]. Genes encoded by SPI4 and SPI1 are coregulated through the master regulator SirA and SPI1-encoded HilA [6,7]. The giant, non-fimbrial adhesin SiiE is secreted by the T1SS and transiently retained on the bacterial surface. SiiE is a linear molecule with a length of 175 ± 5 nm [8,9] that is sufficient to protrude the LPS layer of the outer membrane. The C-terminal moiety of SiiE mediates the first contact to the host cell apical membrane and this function may allow the proper positioning of the SPI1-encoded T3SS.

The non-fimbrial, BIg domain-containing adhesin SiiE initiates the interaction of *Salmonella* with host cells. This review focuses on structure, function and binding properties of this outstanding protein that is involved in *Salmonella* adhesion to and invasion of polarized epithelial cells.

## 2. SiiE—A Non-Fimbrial Adhesin of *Salmonella enterica*

### 2.1. Role of Non-Fimbrial Adhesins in Salmonella Pathobiology

**Figure 1:**
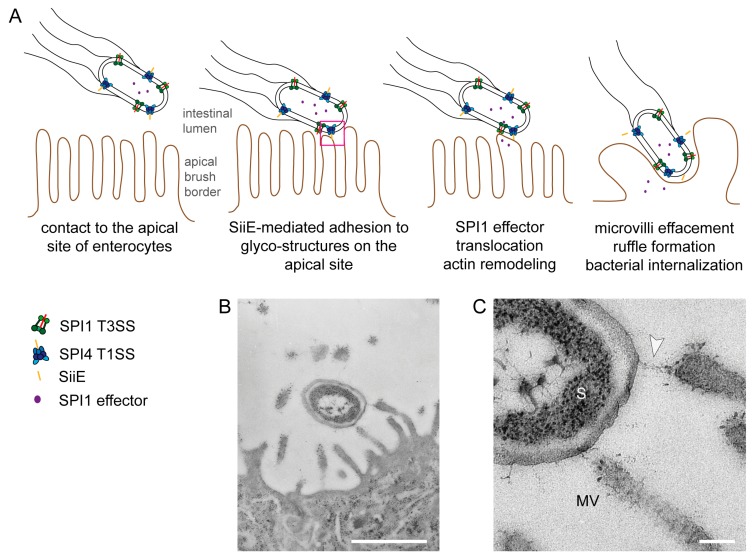
Model for the cooperation of the *Salmonella* Pathogenicity Island 4-encoded type I secretion system (SPI4-T1SS) and the *Salmonella* Pathogenicity Island 1-encoded type III secretion system (SPI1-T3SS) during invasion of polarized cells by *S. enterica*. (**A**) The apical side of polarized epithelial cells has a dense array of microvilli (i); The SPI4-encoded T1SS secretes SiiE that mediates adhesion and intimate contact of *Salmonella* to the apical membrane (ii); This close contact is required for the efficient translocation of the effector proteins by the SPI1-T3SS (iii); SPI1-T3SS effector proteins remodel the host cell actin cytoskeleton, resulting in formation of membrane ruffles and internalization of *Salmonella* and in the effacement of the brush border (iv). In the absence of the SPI4-T1SS and the adhesin SiiE, contacts between bacteria and host cells are highly reduced and translocation of SPI1-T3SS effectors is inefficient. Consequently, these mutant strains are severely compromised in host cell entry from the apical side (adopted from [5]); (**B**,**C**) Ultrastructure of *Salmonella* interaction with the apical side of MDCK cells during infection. Infection of the apical side of MDCK cells was performed using a MOI of 10, cells were fixed 30 min post infection and further processed for transmission electron microscopy (TEM). Close up view of the contact site between *Salmonella* and the host cell. The arrowhead indicates a putative SiiE-mediated connection. S = *Salmonella*, MV = microvillus. Scale bars, 1 µm (B), 100 nm (C). Reproduced, with permission, from Ref. [8].

As a prerequisite for host colonization, *Salmonella* is equipped with a large number of adhesive structures. Besides fimbrial adhesins like type 1 fimbriae or Curli, *Salmonella* possesses the autotransported adhesins MisL and ShdA, and T1SS-secreted adhesins BapA and SiiE [10]. Many of these adhesins are encoded within *Salmonella* Pathogenicity Islands (SPI). For example, the T1SS-secreted adhesin BapA is encoded by a gene within SPI9 and contributes to biofilm formation [11]. The autotransported adhesins MisL and ShdA are encoded by genes within SPI3 and the CS54 island, respectively. Expression of both adhesins is induced in the murine intestine, where they contribute to intestinal persistence [12,13]. Both adhesins, MisL and ShdA, show binding to fibronectin [14,15]. The giant non-fimbrial adhesin SiiE is encoded by SPI4 [16]. SPI4 gene expression is regulated by the transcriptional activator HilA, which is controlled by the master regulator SirA [6,7,17]. SPI4 genes are transcribed into a very long transcript of 27 kb. An operon polarity suppressor (*ops*) is located within the promoter region [18] and this regulatory element recruits the anti-termination factor RfaH to ensure transcription also of distal genes in long operons [19]. Genes in SPI1 are also regulated by SirA and HilA, indicating tight co-regulation of both loci [7]. Indeed, both SPI1 and SPI4 are absolutely necessary for invasion of polarized epithelial cells by *Salmonella* [5]. The current working model proposes that SiiE mediates the first contact between *Salmonella* and polarized host cells (Figure 1). Subsequently, the needle of the SPI1-T3SS can establish contact to the host cell membrane and the translocation of a cocktail of effector proteins into the host cell is initiated. These effector proteins lead to massive rearrangement of the host cell actin cytoskeleton, resulting in microvilli effacement, membrane ruffle formation and finally, the uptake of *Salmonella* [5].

### 2.2. Non-Fimbrial Adhesins

Two major classes of adhesins are distinguished, *i.e.*, fimbrial and non-fimbrial adhesins, which differ in their structure and assembly pathways [20]. Fimbrial adhesins are more common than non-fimbrial adhesins among Gram-negative bacteria. Many of them bind to glycostructures on the host cell membrane. For example, FimH, located at the tip of type I fimbriae, binds to mannose-containing structures [21] and the P-fimbrial tip adhesin PapG binds to Gal α(1,4) Gal [22]. Assembly pathways for fimbrial adhesins are, for example, the chaperone-usher pathway, the extracellular nucleation pathway, and a type II secretion system related mechanism for type IV pili [10]. Fimbrial adhesins are characterized by their straight and rigid structure. They are polymers composed of several subunits and mediate adhesion to host cell structures.

In contrast, non-fimbrial adhesins are commonly mono- or oligomers of a single protein subunit that may be either cell-associated or secreted. Non-fimbrial adhesins are not assembled by the chaperone-usher pathway. One subset of non-fimbrial adhesins is secreted by the autotransporter pathway, also termed type V secretion system (T5SS) [23]. The *Yersinia* adhesin YadA is one of the best characterized trimeric autotransported adhesins (TAA) [24]. Like BadA from *Bartonella henselae* YadA binds to ECM proteins like fibronectin or collagen [25,26]. TAA are characterized by a “lollipop” containing head group, harboring the adhesion function, the neck, the stalk and the highly repetitive fibers.

Another subset of non-fimbrial adhesins is secreted by T1SS. T1SS are composed of three subunits: the ATP-binding cassette protein (ABC), the periplasmic adapter protein (PAP) and the outer membrane protein (OMP) [27]. The largest T1SS-secreted adhesin is LapA from *P. fluorescens* with a size of 900 kDa [28]. The *S. enterica* genome encodes two T1SS-secreted non-fimbrial adhesins; BapA and SiiE. With 595 kDa, SiiE is the largest protein of the *Salmonella* proteome [11,16].

SiiE, like many other non-fimbrial adhesins binds to glycostructures at the cell surface. The two *Helicobacter pylori* adhesins BapA and SabA bind Lewis B antigens and sialic acid, respectively [29,30]. For BapA, it was recently published that there are multiple bonds between the adhesin and the Lewis B antigen [31].

### 2.3. The BIg Domain in Bacterial Proteins

BIg domains consist of Immunoglobulin (Ig) domains. Ig domains are structural motifs in proteins and are part of the Immunoglobulin Superfamily (IgSF). Common features of Ig domains are the domain size of at least 100 amino acids, the number of strands, and the strand topology [32,33]. Ig domains are composed of about 7–10 β-strands, whereupon two strands show typical topology and connectivity [34]. Structural analysis revealed also additional strands and helices as being part of the classical scaffold [34].

Proteins containing Ig domains are not only abundant in eukaryotes, but are also distributed among other domains of life. They can be separated into sixty different groups, with some being more abundant than others. Ig-folds present in Fibronectin type III (Fn3) and cadherin domains are two of the most abundant forms. Fn3 domains are also found in bacterial proteins. So-called bacterial Ig domains (BIg) were identified in bacteria, archaea and bacteriophages. Until now, there is no certain definition for BIg domains, but BIg containing proteins are often associated with bacterial virulence or adhesion to surfaces and membranes.

Especially bacterial adhesins belonging to the Bap family, which are involved in biofilm formation, are composed of BIg repeats. Bap proteins are rather large and found among various Gram-positive and Gram-negative bacteria, like Bap from *Staphylococcus aureus*, LapA from *Pseudomonas fluorescens* or BapA from *Salmonella enterica* [35]*.* Other surface-located or secreted BIg domain-containing adhesins are Invasin from *Yersinia pseudotuberculosis* [36,37] and SiiE from *Salmonella enterica* [8]. The mucus-binding protein (MUB) of the non-pathogenic, Gram-positive bacterium *Lactobacillus reuteri* also shows similarity to Ig-binding proteins [38]. It possesses in total 14 MUB repeats, six MUB1 and eight MUB2 repeats [39]. BIg-domain containing adhesins show strong variation in the number of repeats. For Gram-negative bacteria it has been assumed that the number of repeats is correlated with the thickness of the outer membrane and the O-antigen layer of the LPS [9].

Raman *et al.* reported that BIg domains are capable to bind Ca^2+^ ions [40]. Leptospiral immunoglobulin-like (Lig) proteins LigA and LigB bind Ca^2+^ ions in a structural manner, probably promoting interaction of Lig with extracellular matrix proteins. Ca^2+^ ion binding is also known for SiiE from *S*. *enterica* [9], the MUB2 repeats of MUB from *L. reuteri* [38], SpaA from *Corynebacterium diphtheriae* [41], or the T1SS-secreted hemolysin HlyA from *Escherichia coli* [42]*.* One function of Ca^2+^ ions is stabilization of the protein.

Besides the structural function to bridge the distance between the host cell surface and the bacterial envelope, BIg domains can also bind to host cell receptors. The EPEC protein Intimin binds to the T3SS-translocated receptor Tir. The D3 domain of Intimin is supposed to mediate lectin-like binding [43]. Recently, we have shown that the *Salmonella* non-fimbrial adhesin SiiE also binds in a lectin-like manner to host cell receptors [44].

### 2.4. The Giant Adhesin SiiE

SPI4 genes encode six proteins, termed SiiA-F for “*Salmonella* intestine infection”. SiiC, D and F form a T1SS with SiiF being the ATPase subunit, SiiD the periplasmic adapter protein and SiiC the outer membrane pore. SiiE is the substrate of the T1SS, which is secreted and surface retained during cultivation [8]. Two accessory proteins SiiA and SiiB are present that regulate SiiE surface retention and release, and recent work suggests that SiiAB form a proton channel similar to MotA and MotB of the flagellar motor [45]. The detailed process of SiiE retention and release has still to be investigated.

**Figure 2:**
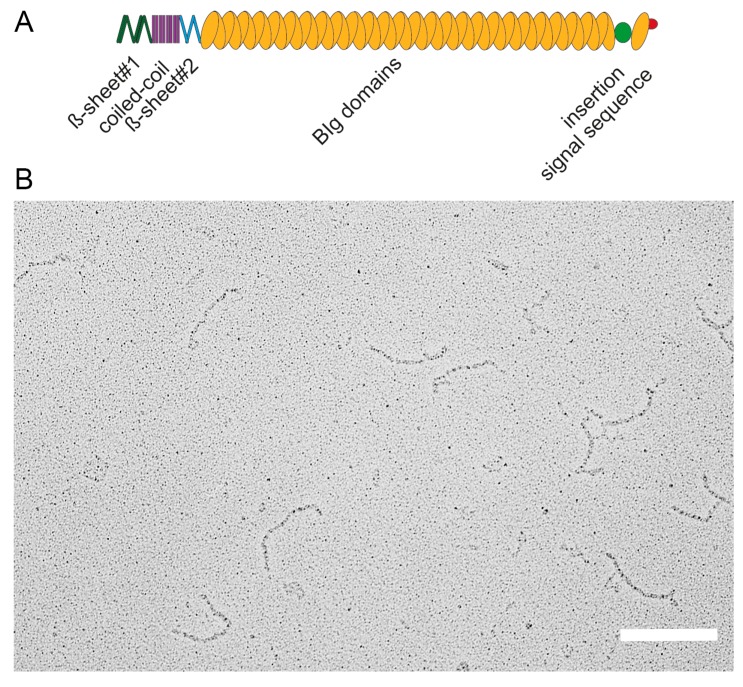
Domain organization and structure of the giant adhesin SiiE. (**A**) Schematic overview of domains within the giant adhesin SiiE. The β-sheet#1, coiled-coil and the β-sheet#2 domains are N-terminally located, followed by 52 bacterial immunoglobulin (BIg) repeats. A 60 amino acid insertion of unknown function is positioned between BIg52 and BIg53. The signal sequence is located to the most C-terminal part; (**B**) Ultrastructure of secreted SiiE. The protein was recovered from the culture supernatant of wild-type *Salmonella* secreting SiiE. SiiE secretion was maximal after 6 h subculture in Luria broth medium aerobically at 37 °C. Scale bar, 200 nm. Reproduced, with permission, from Ref. [8].

The 595 kDa adhesin SiiE is composed of 53 repetitive BIg domains [5,8]. The N-terminus possesses one coiled-coil domain flanked by two β-sheet domains. Between BIg52 and BIg53 a non-BIg domain is located termed “insertion”. The signal sequence for secretion is located in the last C-terminal moiety [8] (Figure 2A). EM analysis of purified secreted SiiE reveals a linear molecular structure of approximately 175 ± 5 nm length [8] (Figure 2B). This length, resulting from a large number of BIg repeats, is essential to protrude the LPS layer. Gerlach *et al.* observed that deletion of an increasing number of BIg domains in SiiE resulted in decreasing polarized cell invasion by *Salmonella* [5]. Deletion of up to five BIg domains could be compensated as SiiE-dependent invasion was present. However, deletion of more than five BIg domains led to a loss of SiiE-dependent invasion [5].

The BIg domains of large non-fimbrial adhesins may be considered as functional homologs of the polymeric shaft subunits of fimbrial adhesins [16]. The polymeric assembly of non-covalently linked subunits in fimbriae allows alteration in the shaft length, but is also more prone to breaking by mechanical forces or disintegration by harsh external conditions. In contrast, large non-fimbrial adhesins consisting of one polypeptide with covalently linked BIg domains will not allow alterations in length, but provide a more robust structure. Despite the presence of a large number of highly repetitive sequences in the BIg repeats [9,16], *siiE* appears genetically stable. Phase variations in SiiE due to frame-shifts, or length alteration by deletion or duplication of BIg repeats were not reported, although in-depth analyses of SiiE expression and structure in collections of *Salmonella* clinical isolates is pending. A remarkable exception is *S. enterica* serovar Typhi and certain other highly host-adapted *S. enterica* serotypes. Due to a frame-shift, *siiE* is a pseudogene in *S*. Typhi [46]. This is in line with the acquisition of pseudogenes in many other genes encoding adhesins in *S*. Typhi and considered as consequence of specialization.

Further dissection of SiiE based on deletion of various domains revealed that the N-terminal moiety is crucial for regulation of retention and release of the protein, whereas the last 60 aa of the C-terminal part harbor the secretion signal and are crucial for SiiE secretion [8]. Mutants with deletions in this part lost the ability to express SiiE on the surface and the invasion into polarized epithelial cells was fully abolished [8]. Moreover, the level of SiiE surface retention is directly correlated with the invasion of polarized epithelial cells. A certain amount of SiiE retained on the bacterial surface is sufficient to mediate invasion [8]. The highest level of SiiE surface retention occurs after 3.5 h subculture in rich media, and also highest SPI1-T3SS-dependent invasiveness was observed in this growth phase. After 6 h of subculture, SiiE was no longer detectable on the bacterial surface but present in the culture supernatant, and adhesion to and invasion of polarized epithelial cells was absent. Certain mutations in SiiE resulted in altered retention and release characteristics. For example, deletion of the β-sheet#2 domain in the N-terminal part of SiiE led to an extended duration of surface retention, but also resulted in a complete loss of SiiE-dependent invasion. Deletions in the β-sheet#1 domain resulted in SiiE over-retention, whereas deletions in the coiled-coil domain led to a decreased retention of the adhesin and SiiE-dependent invasion [8]. These results indicate a role of the coiled-coil domain for SiiE retention and the β-sheet domains for proper release of SiiE that deserve further investigations. In addition to the N-terminus of SiiE, SiiA and SiiB regulate SiiE retention and release by a so far unknown mechanism. The regulation of release of adhesins through external factors is also known for the large adhesin LapA of *Pseudomonas* [47]. At high external P_i_ concentrations, the second messenger c-di-GMP accumulates in the cell and is bound by the effector LapD. c-di-GMP bound LapD sequesters the protease LapG to the inner membrane. This situation allows surface expression of LapA that mediates biofilm formation. In contrast, if low P_i_ concentrations are sensed, LapD is activated, leading to cleavage of c-di-GMP to pGpG. Cellular c-di-GMP is depleted and dissociates from LapD. In turn, LapG is released from the membrane and can cleave the N-terminus of LapA in the periplasm. This proteolysis promotes the release of processed LapA from the cell envelope and return of *Pseudomonas* to planktonic lifestyle [47]. Such proteolytic processing was not observed for SiiE, and protease activity has not been detected in any Sii protein. Rather in line with the controlled SiiE retention by the putative proton channel formed by SiiA and SiiB, increase of the proton-motive force (PMF) by external acidification leads to a higher surface retention of SiiE, whereas PMF destruction by the uncoupler CCCP leads to a dramatic decrease of SiiE surface retention [45].

### 2.5. BIg Domains in SiiE

**Figure 3:**
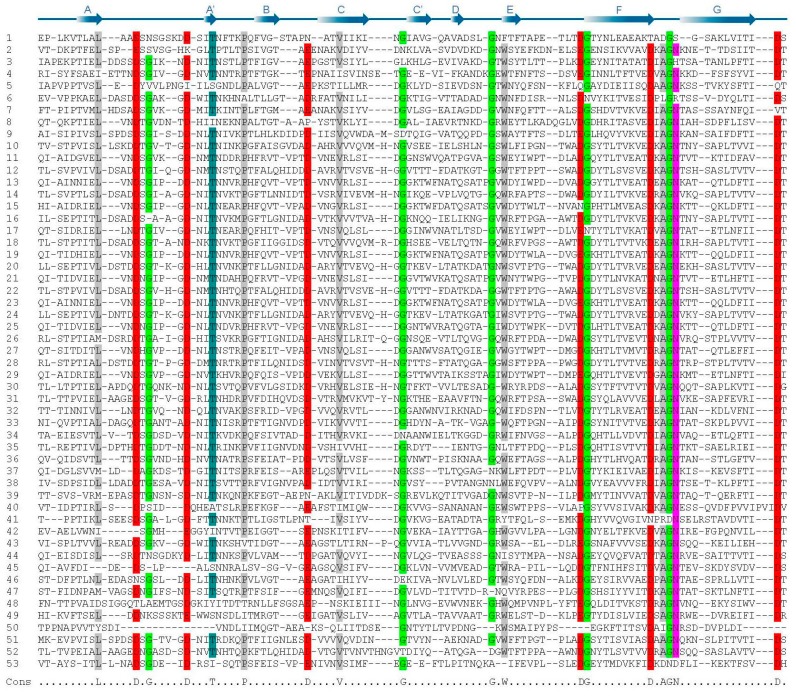
Primary sequence of BIg domains in the giant adhesin SiiE. Multiple sequence alignment of the 53 SiiE BIg domains (UniProtKB accession number Q8ZKG6). Highlighted are conserved amino acids (>80% presence in BIg domains) in red (D or E), light green (G), dark green (T), magenta (N), and gray (A, L, P, V, W). The consensus sequence is shown below the alignment. The scheme above the alignment indicates the β-strand assignment of domain 51 by arrows. Reproduced, with permission, from Ref. [9].

Comparison of primary sequences of the 53 BIg domains in SiiE indicates several residues conserved among all domain repeats (Figure 3). 18 positions exhibit sequence identity of almost 80% in all SiiE BIg domains [16]. For example, six aspartate residues are present, of which ^16^D, ^24^D, ^43^D, ^97^D and ^117^D are involved in Ca^2+^ binding. The additional ^86^D is not involved in Ca^2+^ binding and until now its function remains unclear.

**Figure 4:**
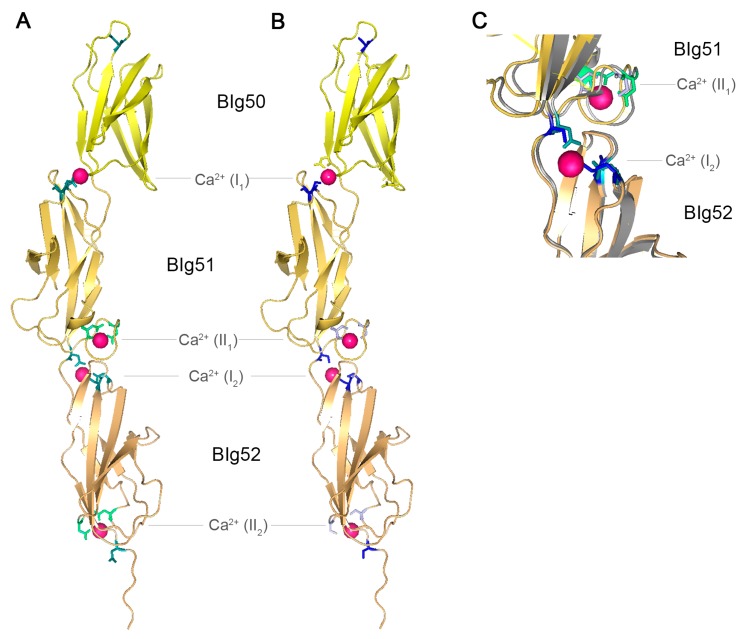
Crystal structure of SiiE BIg50–52. (**A**) Crystal structure of SiiE BIg50–52 with marked conserved aspartate residues. Reproduced, with permission, from Ref. [9]. Conserved D residues of type I Ca^2+^ binding sites are shown in teal, conserved D residues of type II Ca^2+^ binding sites are shown in green. Bound Ca^2+^ ions are depicted in pink; (**B**) Crystal structure of SiiE BIg50–52 with marked conserved aspartate residues exchanged to serine. Conserved D residues of type I Ca^2+^ binding sites and conserved D residues of type II Ca^2+^ binding sites exchanged to S residues are shown in light blue and blue, respectively. Ca^2+^ ions bound by D, but not by S residues, are depicted in pink; (**C**) Close up view and overlay of the type I Ca^2+^ binding site at the interface of BIg51 and BIg52 and of the type II Ca^2+^ binding site of BIg51 and BIg52 with either conserved D residues (teal and green) or mutated S residues (light blue and blue). ^16^D and ^24^D (green and light blue) of BIg51 are involved in the type II Ca^2+^ binding site, whereas ^117^D of BIg52 and 43D and ^97^D (teal and blue) of BIg52 are involved in the type II Ca^2+^ binding site of BIg51. The protein backbone shown in grey belongs to SiiE with conserved D residues, whereas the yellow backbone belongs to SiiE with the D to S exchanges. Ca^2+^ ions bound by D, but not by S residues, are depicted in pink. PyMol was used for the generation of the structure model.

Recently, the crystal structure of SiiE BIg50:52 has been solved [9]. Two types of Ca^2+^ binding sites were identified within SiiE, *i.e.*, type I and type II sites. For type II Ca^2+^ binding sites, ^16^D and ^24^D are residues required. The type I Ca^2+^ binding sites are formed by ^43^D, ^97^D and ^117^D and positioned at the interface between two BIg domains (Figure 4A). Chelating of Ca^2+^ ions results in an increased appearance of kinked conformations of purified SiiE, compared to the elongated rod-like structure under Ca^2+^-bound conditions [9]. Treatment of *Salmonella* with chelating agents reduces the SiiE-dependent invasion into polarized epithelial cells. This indicates a role of Ca^2+^ in stabilizing and rigidifying the protein [9]. Mutational analysis of the conserved D residues also revealed a crucial role of these residues in SiiE release in the culture supernatant (BB, MH; unpublished data). The conserved aspartate residues of type I and type II Ca^2+^ binding sites were exchanged to serine, without influencing the structure of SiiE (Figure 4B,C). The aspartate to serine exchanges prohibit binding of Ca^2+^ ions by SiiE. The net charge of SiiE has not been determined. Since Ca^2+^ ions are complexed within SiiE, the charge depends on the ligands and the ions. For type I Ca^2+^ binding sites with three aspartate residues the net charge will be negative, whereas it is neutral for type II Ca^2+^ binding sites with two aspartate residues. The theoretical isoelectric point (pI) of SiiE is 4.24, indicating more negatively charged residues which can be neutralized through Ca^2+^ binding. Further remarkable sequence identities are in the ^97^D-x-A-G-^101^N motifs, which interlink the type I and type II Ca^2+^ binding sites, and the ^74^W residue. A striking alteration of the canonical BIg domain architecture in SiiE is BIg50. Compared to BIg51 and 52, BIg50 lacks many of the conserved residues. BIg50 only possesses ^97^D, but lacks an additional Ca^2+^ binding site and is also shorter than all other BIg domains in SiiE. So far, the functional relevance of these alterations in BIg50 remains unknown.

Ca^2+^ is also known to control secretion of other T1SS substrates. For the HlyA system of *E. coli* calcium ions stabilize secreted pro-HlyA and facilitate secretion. T1SS substrates are thought to be secreted in an unfolded state, and for some substrates, binding of Ca^2+^ ions can act as a pulling force during secretion by the T1SS [42].

### 2.6. SiiE Binds in a Lectin-Like Manner

Once surface retained, SiiE mediates contact to the apical host cell. Since SiiE-dependent invasion only occurs in polarized epithelial cells like MDCK cells, the target structure of the adhesin could be tissue specific. The organization of the apical membrane of polarized epithelial cells is complex and a dense array of microvilli is present that represents a barrier for invading bacteria. These features may explain the requirement of SiiE for polarized cell invasion. Probably, *Salmonella* needs an additional adhesive mechanism to overcome the microvilli barrier to bind to the host cell. The surface of non-polarized host cells is not structured by microvilli, so other adhesive mechanisms and the binding mediated by the needle of the SPI1-T3SS may be sufficient for invasion.

Various binding specificities for *Salmonella* adhesins have been reported (reviewed in [10]). For example, type 1 fimbriae bind to mannose-containing structures [48], while Std- and Pef-fimbriae bind to α(1–2)-fucosylated proteins and Lewis X blood group antigens, respectively [49,50].

Also other bacterial adhesins bind to glycostructures, like BabA and SabA from *Helicobacter pylori*, which binds LewisB and sialyl LewisA or sialyl LewisX, respectively [51,52,53,54,55]. The type IV pili of *Pseudomonas aeruginosa* bind to asialo GM1 and GM2 [56]. Binding to GlcNAc and α2–3 linked sialic acid containing structures by the giant adhesin SiiE of *Salmonella* was identified through lectin blockade experiments. Various lectins were tested for their capability to block *Salmonella* invasion of polarized and non-polarized epithelial cells. WGA (Wheat germ agglutinin) binding to GlcNAc, and MalII (*Maackia amurensins* lectin II) binding to α2–3 linked sialic acid, led to a dose-dependent decreased invasion into polarized cells. Addition of 25 mg × mL^−1^ of either lectin reduced the invasion of polarized cells to 5% of WT invasion. Since this inhibition was not observable in HeLa cell invasion, this effect was SiiE dependent. Neither addition of α2–6 linked sialic acid, nor α2–6 linked-sialic acid-specific lectin SNA (*Sambucus nigra* agglutinin) were able to block invasion, indicating that the type of hexose, as well as its confirmation within a complex glycostructure are important for recognition by SiiE. The α2–3 linked sialic acid is preferentially localized to the apical side of polarized cells [57], and could act as a receptor for SiiE. Furthermore, GlcNAc and sialic acid are common targets for various microbial adhesins [58].

The requirement for all 53 BIg domains of SiiE in intimate host cell binding is of interest for experimental analysis. Whereas carbohydrate binding by BIg domain proteins is quite common, such binding for non-fimbrial adhesins like SiiE has not been described until now [59]. SiiE is secreted with the C-terminal part first, which carries the secretion signal. Various recombinant SiiE GST fusion proteins with different length were analyzed. A monolayer of polarized epithelial cells was treated with purified proteins, and stained for SiiE. The longer the SiiE fragment was, the more binding was observable [44], indicating that every BIg domain is able to bind the target structure. In line with this is the conserved ^74^W residue [9]. This aromatic residue is conserved in 47 SiiE Blg domains and could be involved in carbohydrate binding [60]. So far, no crystal structure of BIg domains in complex with sugars has been solved. Binding of multiple BIg domains to the target glycan may enhance the affinity for binding. The involvement of Ca^2+^ binding sites in binding of the whole protein to the target structure is also thinkable. Since sialic acid is a negatively charged sugar, the positive charges of the Ca^2+^ ions could also contribute to binding.

The deletion of more than five BIg domains led to decreased SiiE-dependent invasion, indicating that SiiE with 48 or less BIg domains is no longer able to project beyond the LPS layer. According to this model, only a small moiety of SiiE is available outside the LPS layer. Alternatively, the lack of five or more BIg domains contributing to ligand binding may render adhesion ineffective. The more BIg domains bind to glycostructures on the host cell surface, the stronger is the adhesive interaction.

The role of SiiE-dependent invasion *in vivo* is less clear. The initial observation of SiiE function in virulence was made in animal models of *Salmonella* infection. Morgan *et al.* described a role for SiiE in intestinal colonization in cattle, but not in chicken [18,61]. Kiss *et al.* demonstrated that SPI4 is necessary for intestinal but not systemic infections in mice [62]. Using the streptomycin-pretreated mouse model, Gerlach *et al.* showed reduced bacterial burden in cecal lymph nodes after infection with a SPI4-deficient strain. Cecal inflammation was also reduced, but still higher than inflammation scored in mice infected with a SPI1-deficient strain [16]. The complex interaction of pathogenic bacteria and the intestine of the host can only be partially resembled by cell culture or tissue models. For example, mucins are an important component of the intestinal habitat that is usually absent in cell culture models. Mucins are highly glycosylated molecules often containing GlcNAc and sialic acids. It is conceivable that SiiE may also mediate binding of *Salmonella* to mucins during intestinal colonization. However, the presence of functional SiiE in all *Salmonella* serotypes associated with intestinal infections and the tight co-regulation of SPI1-T3SS and SPI4-T1SS function strongly supports an important role of SiiE for life of *Salmonella* in the host intestine.

## 3. Conclusions and Outlook

**Figure 5:**
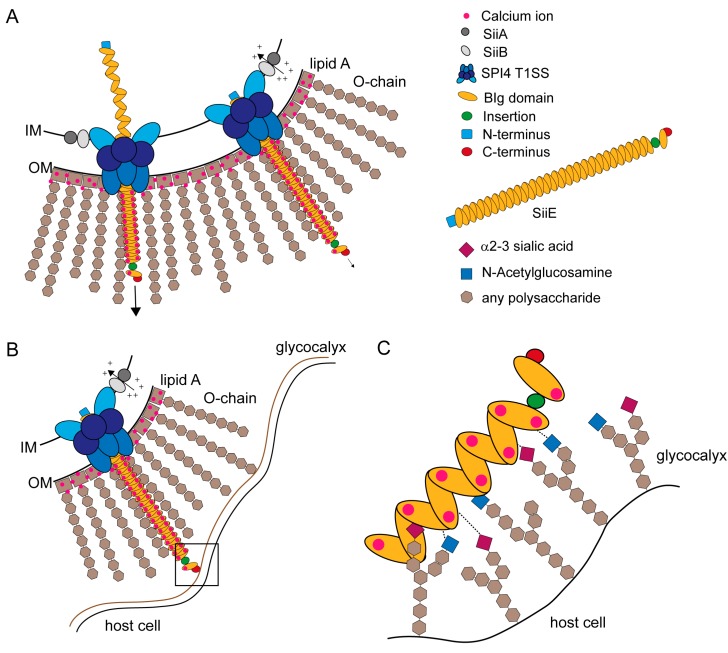
Models for SiiE secretion, surface retention and SiiE-mediated adhesion. (**A**) SiiE is secreted in an unfolded conformation starting with the C-terminus that possesses the secretion signal [63]. Extracellular Ca^2+^ ions, which are present in the LPS layer, bind to conserved D residues and pull SiiE out of the secretion system and facilitate protein folding after secretion (unpublished observations, MH and BB). If SiiE is secreted as far as necessary, the molecule is retained within the secretion system to mediate binding to the host cell surface. Retention is controlled by the accessory proteins SiiA and SiiB. These two proteins form a proton channel that may utilize PMF to energizes SiiE retention or to sense the physiological status of the cell [45]; (**B**) The moiety of SiiE projecting beyond the LPS layer is available for host cell binding. For host cell binding, SiiE is still retained within the secretion system, controlled by SiiA and SiiB [45]; (**C**) Close-up view of the interaction surface (black box in B). Each BIg domain of SiiE is able to bind to glycan-containing structures on the cell surfaces. These structures contain N-acetylglucosamine and α2–3-linked sialic acid [44]. We hypothesize a zipper-like mechanism for SiiE binding that brings the SPI1-encoded T3SS in close proximity to the host cell. The bound Ca^2+^ ions may also facilitate binding to the host cell, since at least sialic acid is negatively charged.

Retention and secretion of the SPI4-encoded non-fimbrial adhesin SiiE is controlled by extracellular Ca^2+^ ions and a proton channel consisting of SiiA and SiiB located in the inner membrane [45] (Figure 5A).

SiiE protrudes the LPS layer with at least five BIg domains [5] to bind GlcNAc and α2,3 linked sialic acid-containing receptors at the apical site of polarized epithelial cells [44] (Figure 5B). SiiE retention during the process of adhesion could be controlled by SiiA and SiiB [45]. Wagner *et al.* [44] showed that there are probably multiple bonds between SiiE and the host cell receptors (Figure 5C). A comparable model was recently described for the *Helicobacter* adhesin BapA [31]. SiiE-mediated adhesion is crucial for *Salmonella* invasion of polarized epithelial cells [5,7]. SiiE-mediated binding is necessary to position the SPI1-encoded T1SS for effector translocation, which lead to bacterial internalization [5].

Further investigations are necessary to understand the whole process of SiiE retention and secretion. What is the signal for SiiE secretion, and when does it occur? Is this signal co-regulated with the SPI1‑T3SS effector translocation or is it only due to extracellular conditions like pH or osmotic stress?

The contribution of each of the BIg domains to ligand binding is also not understood in detail. Most of the BIg domains show high sequence similarities. Yet, BIg50 is much shorter and conserved residues are missing. Also the role of the supposed unstructured domain inserted between BIg52 and BIg53 needs to be elicited. Both of these domains are positioned at the C-terminal part of SiiE, which is supposed to get into contact with the host cell first. A deletion in the insertion domain shows no attenuated phenotype in SiiE retention, secretion or in SiiE mediated invasion [8].

The domain architecture of SiiE, the special mechanism of retention and release, and the strong phenotype in adhesion to, and invasion of polarized epithelial cells clearly call for further in-depth studies of this fascinating bacterial lectin.
